# Association of PCR-based oral bacterial profiles with fluorescence-detected plaque and caries experience in 3-year-old children: a cross-sectional study

**DOI:** 10.1186/s12903-026-07968-6

**Published:** 2026-02-26

**Authors:** Sang-Kyeom Kim, In-Young Ku, Su-Jin Han

**Affiliations:** 1 Department of Preventive Dentistry & Public Oral Health, BK21 FOUR Project, Yonsei University College of Dentistry, Seoul, Republic of Korea; 2 Department of Dental Hygiene, College of Health Science, Kyungwoon University, Gumi, Republic of Korea; 3 Department of Dental Hygiene, College of Medical Science, Gachon University, 191 Hambakmoe-ro, Yeonsu-gu, Incheon, 21936 Republic of Korea

**Keywords:** Dental caries, Oral microbiome, Plaque, Fluorescence imaging, Polymerase chain reaction, Preschool children, Bacteria

## Abstract

**Background:**

Quantitative light-induced fluorescence (QLF) is widely used to detect dental plaque. However, the microbial composition of QLF-detected plaque in young children remains poorly characterized. This cross-sectional study aimed to assess the associations among 10 oral disease-associated bacterial species, QLF-detected plaques, and caries in 3-year-old children.

**Methods:**

Ninety-nine 3-year-old children participated in this study. Real-time polymerase chain reaction (PCR) quantified 10 target species, which were analyzed individually and as functional groups (red-complex, orange-complex, and caries-associated groups). Plaque on the labial surfaces was measured by QLF imaging and scored using the Fluorescence Patient Hygiene Performance Index (F-PHPI). The caries experience (dft) was clinically recorded. Associations were evaluated using Spearman’s correlation and regression analyses.

**Results:**

The F-PHPI score was significantly associated with *Fusobacterium nucleatum* (*F. nucleatum*) (ρ = 0.455, *p* < 0.05), the orange complex (ρ = 0.456, *p* < 0.001), and total bacterial load (ρ = 0.479, *p* < 0.001). Regression analysis showed that orange-complex levels predicted F-PHPI scores (R^2^ = 0.17). dft correlated with the caries-associated group (ρ = 0.282, *p* < 0.05), and logistic regression identified this group as a significant predictor (OR = 1.34, *p* = 0.01), with *Streptococcus mutans* (*S. mutans*) the only individual species associated with caries (ρ = 0.286, *p* < 0.05).

**Conclusions:**

In 3-year-old children, QLF-detected plaque was significantly associated with orange-complex bacteria. The F-PHPI served as a potential indicator of microbial load, with regression models confirming its predictive relationship with pathogenic bacterial groups. These findings support QLF imaging as a noninvasive tool for early microbial risk assessment in pediatric populations.

## Background

Dental plaque is a complex biofilm, and ecological imbalances within it contribute to the risk of oral diseases, depending on the microbial composition and metabolic byproducts [1, 2]. In children, the oral microbiome undergoes dynamic changes during the first few years of life [3–7]. The eruption of primary teeth, which typically occurs between one and two years of age, further increases microbial diversity and abundance, with notable inter-individual variations observed by the age of three [8, 9]. Emerging evidence suggests that the early establishment of the oral microbiome may have long-term effects on overall health [10–14].

Because microbial maturity is associated with oral disease risk, there is a growing interest in visual detection methods that can reflect the pathogenicity of plaques in a noninvasive manner, particularly for use in children. Noninvasive sampling methods, such as saliva collection and mucosal swabbing, are increasingly employed in oral microbiome research involving children [15]. Among these tools, quantitative light-induced fluorescence (QLF) has emerged as a promising technique. When exposed to 405 nm blue light, bacterial metabolites such as protoporphyrin IX emit red fluorescence, enabling real-time visualization of mature dental plaques associated with higher microbial diversity and increased pathogenic potential [16–21].

Previous studies in adults have confirmed that red fluorescence from dental plaque correlates with increased cariogenic and periodontopathogenic potential [22]. For instance, research using 16 S rRNA sequencing and real-time PCR reported significantly greater bacterial diversity and elevated levels of periodontal pathogens at red-fluorescent sites [23, 24]. In contrast, investigations in pediatric populations have tended to examine QLF imaging or microbial profiling in isolation. Studies have either applied QLF primarily to visualize plaque for hygiene education [25] or profiled plaque microbiota using molecular techniques without incorporating fluorescence imaging [26–28]. Consequently, the relationship between the QLF-detected red fluorescence and microbial characteristics in early childhood remains poorly understood. The limited integration complicates the interpretation of QLF signals in preschool-aged children and presents a challenge for its clinical use in microbial risk-based preventive strategies.

Therefore, this study focused on 3-year-old children, a population at a critical stage of oral microbiome development that is particularly well-suited for clinical imaging due to their developmental and behavioral characteristics. This age was also chosen because it represents the youngest group attending daycare and serves as the baseline cohort for a planned longitudinal follow-up at age five. Real-time PCR was used to quantify bacterial DNA in oral plaque samples owing to its high sensitivity and specificity. Ten oral disease-associated bacterial species were selected not only for their pathogenic roles in periodontitis and caries but also as key indicators of early biofilm maturation and dysbiosis [1, 2]. These species are commonly analyzed not only individually, but also as functional consortia, such as the pathogenic complexes associated with periodontal disease or key groups implicated in dental caries. Although severe periodontitis is rare in this age group, monitoring these precursors, such as the orange complex species, provides critical insight into the ecological succession and potential future risk of oral diseases [8].

The first aim of this study was to determine the prevalence and quantity of these 10 bacterial species in 3-year-old children, using real-time PCR. The second aim was to investigate the associations and predictive modeling between bacterial levels, analyzed both at the individual species level and as functional groups broadly categorized by their primary association with either periodontal disease or dental caries, and QLF-detected plaque deposition and caries experience. We hypothesized that higher bacterial levels would be positively associated with greater plaque accumulation, as measured by QLF-based plaque scores, and increased caries experience.

## Methods

### Study subjects and dental examination

This study was conducted as part of an oral health project for preschool children supported by the Seoul Metropolitan Office of Education and Korean Dental Hygienists Association. Ethical approval was obtained from the Institutional Review Board of Gachon University (IRB No. 1044396-202304-HR-052-01). Clinical trial number: not applicable.

A total of 99 Korean children aged three years were recruited through community-based dental health programs at daycare centers across various districts in Seoul. Enrollment was based on voluntary participation following parental informed consent, and random sampling was not applied. The sample size was determined by feasibility and program-based enrollment rather than an a priori power analysis, as all eligible children available during the study period were included. To minimize potential confounding effects on the oral microbiome, children with systemic diseases or those who had taken antibiotics within the last three months were excluded based on parental reports.

All oral examinations were performed by a single dentist who had completed the calibration training for oral health screening. The examinations were conducted in accordance with the World Health Organization (WHO) diagnostic criteria and the oral health survey guidelines. As the study was performed in daycare centers rather than in a clinical setting, a portable dental chair, LED light, and disposable dental mirrors were used. To ensure accuracy, particularly in cases where child cooperation was limited, intraoral video recordings captured via smartphone were reviewed post-examination to verify the clinical data. For each participant, the number of decayed (d) and filled (f) primary teeth was recorded, and the dft (total number of decayed and filled teeth) and dt (number of untreated decayed teeth) were calculated.

### Sample collection

Sampling was performed within one hour of brushing teeth. During this period, participants were instructed to abstain from eating, drinking, or chewing gum. Using a sterile oral swab, supragingival plaque was collected from the buccal and lingual surfaces of all erupted primary teeth in the maxilla and mandible along the gingival margins. The swab was immediately placed into a conical tube containing 10 mL of a commercially available mouthwash solution (Garglin^®^ Original, Dong-A Pharm, Seoul, Korea), which contains approximately 8% ethanol. The sample was then vortexed for 10 s (Vortex Genie 2; Scientific Industries, New York, NY, USA), and the swab was pressed against the tube wall to release any residual fluid before disposal. This method followed the protocol recommended by an external analysis agency (Denomics, Seoul, Korea), and has been validated in prior research as an effective means of preserving microbial diversity in oral samples. Ethanol-containing mouthwash solutions have been widely used in oral microbiome research and are considered suitable for DNA-based bacterial analyses [29, 30].

### Real-time PCR analysis

#### Amplification protocol

Genomic DNA was extracted from 500 µL of the mouthwash suspension using the QIAamp DNA Mini Kit (QIAGEN, Hilden, Germany), following the manufacturer’s instructions. Subsequently, 180 µL of ATL buffer and 20 µL of Proteinase K were added to the sample and incubated at 56 °C for at least one hour. Subsequently, 200 µL of AL buffer was added, followed by incubation at 70 °C for 10 min and the addition of 200 µL of ethanol (96–100%). The lysate was then transferred to a spin column, washed with AW1 and AW2 buffers, and eluted in 200 µL of AE buffer. DNA concentration and purity were assessed using a NanoDrop™ Eight spectrophotometer (Thermo Fisher Scientific, Waltham, MA, USA).

Real-time PCR was performed using species-specific primers and TaqMan probes targeting 10 bacterial species implicated in oral diseases. This selection was based on previous studies that employed similar bacterial panels to assess oral disease risk and characterize plaque-associated microbial communities in children: *Aggregatibacter actinomycetemcomitans* (*A. actinomycetemcomitans*), *Porphyromonas gingivalis* (*P. gingivalis*), *Tannerella forsythia* (*T. forsythia*), *Treponema denticola* (*T. denticola*), *Prevotella intermedia* (*P. intermedia*), *Fusobacterium nucleatum* (*F. nucleatum*), *Prevotella nigrescens* (*P. nigrescens*), *Streptococcus mutans* (*S. mutans*), *Streptococcus sobrinus* (*S. sobrinus*), and *Lactobacillus casei* (*L. casei*) [31, 32]. Probes were synthesized by Macrogen (Seoul, Korea), labeled with fluorescent dyes (FAM, HEM, Texas Red, or Cy5), and quenched with BHQ1 or BHQ2. Each 20 µL PCR reaction mixture consisted of 5 µL of DNA template, 0.3 µL of forward primer (1.5 pmol/µL), 0.05 µL of reverse primer (0.25 pmol/µL), 0.3 µL of probe (1.5 pmol/µL), 10 µL of 2× qPCR Master Mix (Genet Bio, Daejeon, Korea), and nuclease-free water. Amplification was performed using a CFX96 Real-Time PCR Detection System (Bio-Rad, Hercules, CA, USA), with the thermal cycling conditions set at 95 °C for 10 min for initial denaturation, followed by 45 cycles of 95 °C for 15 s and 60 °C for 60 s.

#### Quantification and bacterial group classification

Bacterial load was quantified using a universal primer targeting the 16 S rRNA gene. The total bacterial number, defined as the number of 16 S rRNA gene copies per 10 mL of mouthwash, served as a reference for evaluating bacterial group composition. The 10 target species were grouped functionally according to their pathogenic potential and clinical associations with oral diseases. The red and orange complexes were classified according to their established roles in periodontal pathogenesis [33]. However, the caries-associated group was defined in this study to reflect the species commonly detected in early childhood. *L. casei* was included based on its consistent detection in carious dentin and prevalence among lactobacilli isolated from carious lesions in both children and adults [34, 35]. Specifically, *P. gingivalis*, *T. forsythia*, and *T. denticola* (red complex) were designated as high-risk periodontal pathogens, *P. intermedia*, *F. nucleatum*, and *P. nigrescens* (orange complex) formed the moderate-risk group, and *S. mutans*, *S. sobrinus*, and *L. casei* comprised the caries-associated group. For each group, the total copy number was calculated by summing the copy numbers of individual species.

### QLF Imaging and fluorescence-based plaque assessment

QLF images were obtained using a Qraycam Pro (AIOBIO, Seoul, Korea), a clinical-grade intraoral fluorescence imaging system. Only frontal images of the maxillary and mandibular anterior teeth were obtained due to the participants’ young age and limited cooperation. A real-time video was recorded on a PC, and optimal still frames were extracted for analysis.

**Fig. 1:**
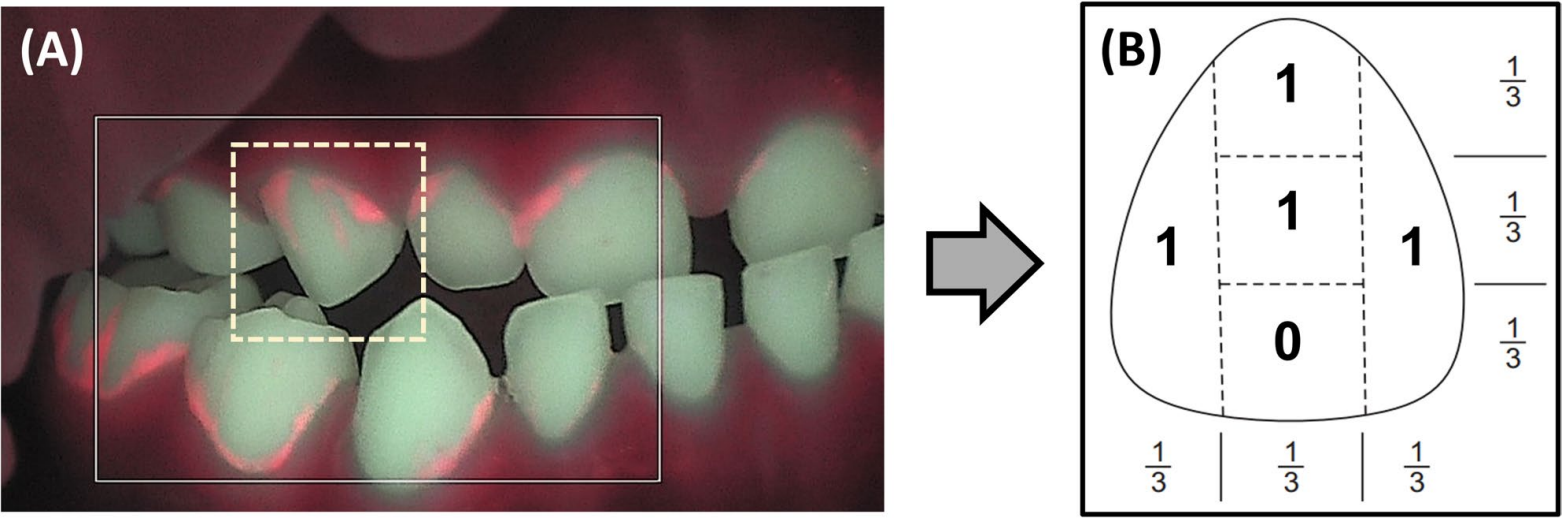
**A** Representative QLF image of anterior primary teeth captured from a fluorescence video using the Qraycam Pro. Red fluorescence indicates areas of mature plaque. **B** Schematic representation of the F-PHPI scoring method. The labial surface of each anterior tooth is divided into five segments (gingival, middle, incisal, mesial, and distal), each scored as 1 (presence of red fluorescence) or 0 (absence). The total score per tooth ranges from 0 to 5

To quantify plaque accumulation, a modified scoring system was developed based on the Patient Hygiene Performance Index (PHPI) originally proposed by Podshadley and Haley [36] (Fig. 1). In this fluorescence-based modification (F-PHPI), each labial surface of the 12 anterior primary teeth (central and lateral incisors and canines) was divided into five segments: gingival, middle, incisal, mesial, and distal. Segments exhibiting red fluorescence were assigned a score of 1, whereas those without fluorescence were assigned a score of 0. A tooth-level score ranging from 0 to 5 was calculated and the average score across all 12 teeth was used to derive the F-PHPI score for each participant.

While the F-PHPI was designed to provide a structured scoring of red fluorescence in anterior primary teeth, formal validation procedures (e.g., reliability testing or comparison with conventional indices) were not conducted in this study.

### Statistical analysis

All statistical analyses were conducted using Python, version 3.13.2 (Python Software Foundation) and its associated scientific computing libraries including Pandas (2.2.3), NumPy (2.2.5), SciPy (1.15.3), and Statsmodels (0.14.5).

Bacterial copy numbers for each of the 10 target species were log_10_(x + 1)-transformed to stabilize variance, and these transformed values were summed to obtain totals for the red complex, orange complex, a newly defined caries-associated group (*S. mutans*, *S. sobrinus*, *L. casei*), and the total bacterial load.

To assess non-parametric associations between log_10_(x + 1)-transformed bacterial counts and clinical indices (F-PHPI, dft, and dt), Spearman’s rank correlation was used, and the Benjamini–Hochberg false discovery rate (FDR) correction was applied for multiple testing. To further evaluate the associations of primary interest, simple linear regression was used for continuous clinical indices, and simple logistic regression was used for binary outcomes. For non-parametric group comparisons, Mann-Whitney U tests were applied after dichotomizing the F-PHPI scores at the sample median and defining caries experience as dft > 0. Differences were considered statistically significant at FDR-adjusted *p* < 0.05.

## Results

### Demographic and oral health characteristics of participants

A total of 99 three-year-old children (43 boys, 56 girls) participated in this study. The overall dft rate, defined as the proportion of children with at least one decayed or filled primary tooth, was 17%, and the dft index, representing the average number of decayed or filled teeth per child, was 0.57 ± 1.78. The average F-PHPI score, which reflects red fluorescent plaque deposition on the labial surfaces of the 12 anterior primary teeth, was 1.15 ± 0.98.

### Prevalence and copy number of oral bacterial species

**Fig. 2:**
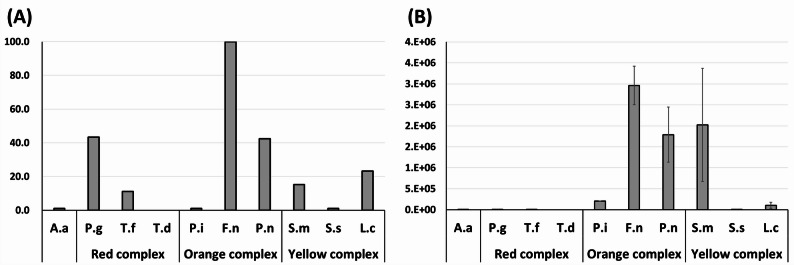
**A** Prevalence (%) of oral pathogenic bacteria among participants and **B** bacterial copy numbers (mean ± SD) of the target species. A.a, *Aggregatibacter actinomycetemcomitans*; P.g, *Porphyromonas gingivalis*; T.f, *Tannerella forsythia*; T.d, *Treponema denticola*; P.i, *Prevotella intermedia*; F.n, *Fusobacterium nucleatum*; P.n, *Prevotella nigrescens*; S.m, *Streptococcus mutans*; S.s, *Streptococcus sobrinus*; L.c, *Lactobacillus casei*

Among the 10 target bacterial species, *F. nucleatum* was detected in all participants (100%), followed by *P. gingivalis* (43.4%), *P. nigrescens* (42.4%), and *L. casei* (23.2%). *S. mutans* and *T. forsythia* had a prevalence of 15.2% and 11.1%, respectively. The lowest prevalence (1.0%) was recorded for *A. actinomycetemcomitans*, *P. intermedia*, and *S. sobrinus* (Fig. 2A).

Based on absolute bacterial copy numbers, *F. nucleatum* exhibited the highest levels, followed by *S. mutans*, *P. nigrescens*, *P. intermedia*, and *L. casei*. Conversely, the red-complex species (*A. actinomycetemcomitans*, *P. gingivalis*, *T. forsythia*, and *T. denticola*) showed consistently low copy numbers across the participants (Fig. 2B).

### Correlations of bacterial profiles with plaque index and caries experience

Spearman’s rank correlation analysis revealed significant associations between specific bacterial species and the clinical indices after FDR adjustment (Table 1). The F-PHPI score demonstrated a significant positive correlation with the copy numbers of *F. nucleatum* (ρ = 0.455, *p* < 0.05). *S. mutans* was positively correlated with dft (ρ = 0.286, *p* < 0.05) and dt (ρ = 0.298, *p* < 0.05).

**Table 1:** Spearman’s rank correlations between individual bacterial species and clinical indices

Bacterial species	dt	dft	F-PHPI
*A. actinomycetemcomitans*	−0.036	−0.044	n.c.
*P. gingivalis*	−0.128	−0.049	0.068
*T. forsythia*	0.087	0.040	0.063
*T. denticola*	n.c.	n.c.	n.c.
*P. intermedia*	−0.036	−0.044	0.139
*F. nucleatum*	−0.201	−0.195	0.455*
*P. nigrescens*	0.039	0.052	0.237
*S. mutans*	0.298*	0.286*	0.313
*S. sobrinus*	−0.036	−0.044	−0.158
*L. casei*	0.119	0.159	0.059

Spearman’s rank correlation coefficients (ρ) with p-values adjusted for multiple testing by the Benjamini–Hochberg false discovery rate (FDR) are presented. n.c., not computed because the bacterial data showed no variance owing to the very low prevalence of the species

*dt* number of untreated decayed primary teeth, *dft* number of decayed or filled primary teeth, *F-PHPI* fluorescent Patient Hygiene Performance Index

*p < 0.05

When bacteria were analyzed as functional groups (Table 2), these associations became more pronounced. For plaque accumulation, correlations were stronger for the orange complex (ρ = 0.456, *p* < 0.001) and for total bacterial load (ρ = 0.479, *p* < 0.001) than for any single species. For caries experience, a significant association was observed between dft and the caries-associated group (ρ = 0.282, *p* < 0.05).

**Table 2:** Spearman’s correlation between bacterial groups and clinical indices

Bacterial group	dt	dft	F-PHPI
Red complex	−0.099	−0.034	0.077
Orange complex	−0.176	−0.173	0.456***
Caries-associated group	0.230	0.282*	0.151
Total bacterial load	−0.158	−0.157	0.479***

Spearman’s rank correlation coefficients (ρ) with p-values adjusted for multiple testing by the Benjamini–Hochberg false discovery rate (FDR) are presented

*dt* number of untreated decayed primary teeth, *dft* number of decayed or filled primary teeth, *F-PHPI* fluorescent Patient Hygiene Performance Index

*p < 0.05, **p < 0.01, ***p < 0.001

### Regression and group-comparison analyses of key bacterial associations

To further evaluate the two major associations identified in the correlation analysis, the link between the orange complex and QLF-detected plaque and that between the caries-associated group and caries experience, regression analyses were performed (Table 3). In the linear model, the log_10_(x + 1)-transformed load of the orange complex significantly predicted the F-PHPI score (β = 0.458, *p* < 0.001), explaining 17.0% of the variance (R^2^ = 0.170). Logistic regression showed that the caries-associated group significantly predicted caries experience (dft > 0), with each 10-fold increase in bacterial load associated with a 1.34-fold increase in the odds of having caries (OR = 1.34, 95% CI: 1.07–1.68, *p* = 0.01; pseudo-R^2^ = 0.077).

**Table 3:** Regression analyses of bacterial groups and clinical outcomes

Clinical outcome	Predictor (log_10_[x + 1])	β/OR (95% CI)	*p*-value	Model fit
F-PHPI (linear)	Orange complex	0.458 (0.249, 0.666)	< 0.001	R^2^ = 0.170
Caries experience (logistic)*	Caries-associated group	1.34 (1.07, 1.68)	0.01	Pseudo-R^2^ = 0.077

*F-PHPI* Fluorescent Patient Hygiene Performance Index, *β* regression coefficient for linear model, *OR* Odds ratio for logistic model

Bacterial copy numbers were log_10_(x + 1)-transformed

P-values were two-sided and unadjusted for multiple testing

*Caries experience, defined as dft > 0. Odds ratio (OR) derived from the logistic regression coefficient

**Fig. 3:**
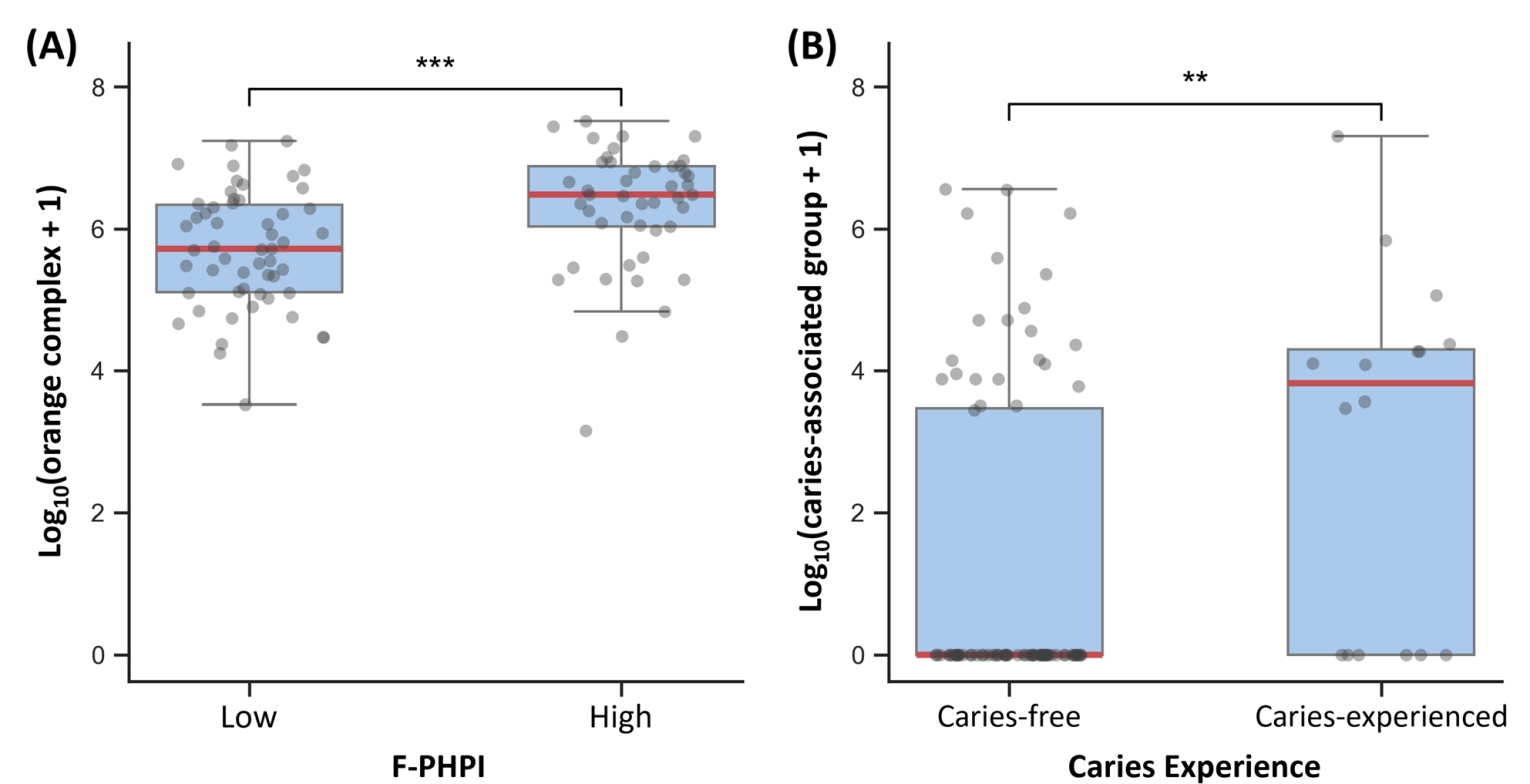
Boxplots showing the distribution of log_10_(x + 1)-transformed bacterial loads between clinical groups The central line indicates the median, the box represents the interquartile range (IQR), and whiskers extend to 1.5 × IQR; individual data points are overlaid as semi-transparent dots. **A** Orange complex loads in high and low plaque groups, defined by the median F-PHPI score. **B** Caries-associated group loads in children with (dft > 0) and without caries experience. Statistical significance was assessed by the Mann–Whitney U test and is denoted by asterisks. F-PHPI, Fluorescence Patient Hygiene Performance Index. **p < 0.01, ***p < 0.001

These relationships were also evident in the box plots (Fig. 3). Children in the high-plaque group, defined by an F-PHPI score above the sample median, exhibited significantly higher orange complex loads than those in the low-plaque group (Mann-Whitney U test, *p* < 0.001; Fig. 3A). Likewise, children with caries experience showed higher bacterial loads of the caries-associated group than those in the caries-free group (*p* < 0.01; Fig. 3B).

## Discussion

This study demonstrated that QLF-detected red fluorescent plaque in three-year-old children was significantly associated with the orange complex, an association primarily driven by *F. nucleatum*. In parallel, caries experience was positively related to the caries-associated group, with *S. mutans* emerging as a key individual contributor (Tables 1 and 2). These relationships were consistently supported by the correlation, regression, and group-comparison analyses (Table 3; Fig. 3). To the best of our knowledge, no prior study has combined QLF-based fluorescence imaging with quantitative profiling of disease-associated species in preschool populations. While previous studies have applied QLF imaging in school-aged children to visualize plaque and support hygiene education [25], and other studies have profiled the microbial composition of pediatric plaque using molecular techniques without incorporating QLF [26–28], no prior research has combined fluorescence imaging and microbial quantification in a preschool population. This distinction underscores the novelty of the current study and its contribution to early stage characterization of biofilm maturity and oral disease risk. These findings may serve as a foundational reference for future research on oral pathogens in preschool populations and support the application of fluorescence-based approaches to evaluate pediatric oral hygiene.

*A. actinomycetemcomitans* and members of the red complex (*P. gingivalis*, *T. denticola*, and *T. forsythia*) are well-known keystone pathogens in adult periodontitis [37, 38]. In the present study, *P. gingivalis* was detected in approximately 43% of participants, yet the absolute copy numbers of all red complex species were consistently low (Fig. 2B). These low bacterial levels likely represent the physiological establishment of early colonizers characteristic of a healthy pediatric population, rather than active periodontal destruction, which is consistent with the low prevalence of periodontitis in young children and its increasing prevalence with age [39]. Monitoring these species in early childhood is nonetheless valuable, as their establishment may indicate the initiation of ecological succession, potentially predisposing individuals to a higher risk in later life [40].

In the current study, orange complex bacteria, most notably *F. nucleatum*, were significantly associated with plaque accumulation levels as detected by QLF (Tables 1 and 2). Consistent associations were confirmed in subsequent regression and group-comparison analyses (Table 3; Fig. 3). This finding is clinically relevant because the orange complex serves as a key ecological link between early and late colonizers within dental plaque biofilms [38]. It is well established that red complex species are rarely found in the absence of orange complex bacteria, and that increases in orange complex colonization are often accompanied by a corresponding rise in red complex colonization [37]. In this study population, the universal detection of *F. nucleatum* may reflect early microbial colonization and biofilm maturation rather than established disease processes. While *F. nucleatum* is also studied for its systemic implications [41–43], its primary relevance in this preschool context lies in its contribution to the ecological shift toward a more cariogenic and periodontopathogenic plaque profile. Therefore, the significant correlation between red fluorescence and orange complex species suggests that F-PHPI scores may effectively screen for biofilm environments conducive to pathogenic succession, serving as an early indicator of microbial dysbiosis.

Bacteria in the caries-associated group, including *S. mutans*, *S. sobrinus*, and *L. casei*, are well established as key contributors to dental caries, particularly in early childhood. Although these species can also be detected in individuals with clinically healthy periodontal tissues [33], their elevated presence, especially in terms of bacterial copy number, has consistently been associated with an increased caries risk. Several PCR-based studies have reported that children harboring both *S. mutans* and *S. sobrinus* exhibit a higher prevalence of caries and greater dmft scores than those colonized by only one species [44, 45]. Similarly, *Lactobacillus* spp., including *L. casei*, are more abundant in children with severe early childhood caries, with bacterial levels positively correlating with caries experience [46]. In this context, the findings of the present study align with those of previous studies, showing a significant positive correlation between the caries-associated group and caries experience (Table 2). This association was further supported by group comparisons, which showed that children with caries had significantly higher loads of this bacterial group than their caries-free counterparts (Fig. 3B). Logistic regression analysis quantified this risk, revealing that an increased load in the caries-associated group was a significant predictor of caries (Table 3).

This study suggests the potential of the QLF technology as a tool for evaluating oral hygiene in children. Unlike conventional indices such as the Patient Hygiene Performance (PHP) index, which requires disclosing agents, the fluorescence-based F-PHPI introduced in this study may facilitate efficient, non-invasive assessment of plaque accumulation on the labial surfaces of anterior teeth. This approach could be particularly appropriate for young children, for whom conventional plaque scoring may be less feasible. Notably, this simplified imaging of the anterior labial surfaces alone captured microbial patterns that reflected the whole-mouth plaque status, highlighting its practicality for rapid screening in pediatric settings. Beyond serving as a basic oral hygiene assessment tool, the F-PHPI may also function as an early indicator of disease risk, owing to its association with pathogenic microorganisms. In particular, its significant correlation with orange complex bacteria (Table 2), which play a key role in microbial succession and disease progression, underscores the clinical significance of the index. This association was further confirmed by regression analysis, which showed that the orange complex strongly predicted F-PHPI scores (β = 0.458, R² = 0.170; Table 3). Red fluorescence-based QLF imaging may therefore offer a practical, non-invasive approach not only for assessing oral hygiene but also for anticipating future oral health outcomes in children.

This study had several limitations. First, standard clinical indices for gingival health (e.g., gingival index) or oral hygiene performance (e.g., plaque index using disclosing agents) were not recorded. This limits the direct comparison between microbial profiles and clinical periodontal status. However, this study aimed to evaluate QLF as a non-invasive screening tool for early risk assessment in community settings where periodontal probing or plaque staining is impractical for young children. Second, data collection was constrained by challenges inherent to the pediatric population and the non-clinical daycare setting, which, while reflecting real-world conditions, limited the range of clinical data that could be collected. Limited cooperation and a non-clinical environment necessitated confining QLF imaging to the anterior teeth, whereas microbial samples were collected via whole-mouth swabbing. This anatomical mismatch implies that the F-PHPI scores may not fully capture the greater microbial burden typically found in posterior stagnation areas, potentially attenuating the observed correlations. However, a previous study reported that fluorescence of anterior dental biofilms is significantly correlated with whole-mouth clinical indices, including the plaque index (*r* = 0.499) and gingival index (*r* = 0.422) [47]. This indicates that anterior fluorescence may reflect overall plaque and gingival conditions, partially supporting the use of anterior QLF imaging as a surrogate for comprehensive plaque assessment in field-based pediatric settings. Third, although the F-PHPI was adapted from an established plaque index [36], it served as an exploratory measure in this study. As this modified index has not yet undergone formal validation or reliability testing, the findings regarding the F-PHPI should be interpreted with caution. Additionally, the statistical analysis relied primarily on bivariate correlations, complemented by exploratory regression and group-comparison analyses. Potential confounding variables were not adjusted for, partly because the non-clinical setting and limited data collection inherent to this pediatric study restricted the availability of covariates within this exploratory framework. Given these constraints and the cross-sectional design, the observed associations between bacterial profiles and F-PHPI scores should be interpreted cautiously, as they represent correlational relationships rather than causal effects.

Despite these limitations, this study offers a novel contribution by integrating a QLF-based fluorescence assessment with microbial profiling in preschool-aged children. Future studies should aim to validate the F-PHPI, incorporate posterior tooth imaging, and apply multivariate statistical models using comprehensive behavioral and clinical data. Site-specific plaque sampling may also improve the anatomical concordance between imaging findings and microbial profiles. Longitudinal follow-ups are essential to clarify how early microbial colonization and plaque fluorescence are related to future oral and systemic health. An ongoing oral health programme involving this cohort may serve as a valuable platform for future investigations.

## Conclusions

These findings suggest that the newly developed QLF-based F-PHPI shows promise as a potential non-invasive screening tool for monitoring biofilm maturation and oral disease risk in young children. The F-PHPI demonstrated significant associations with disease-related bacterial groups, which were further supported by regression analyses, suggesting its potential role as an early microbial risk indicator. To confirm the reliability of this exploratory index and support its broader clinical and public health applications, further longitudinal studies and formal validation against established clinical indices are necessary.

## Data Availability

The datasets used and/or analyzed during the current study are available from the corresponding author on reasonable request.

## Abbreviations

F-PHPI: Fluorescence Patient Hygiene Performance Index
PCR: Polymerase chain reaction
QLF: Quantitative light-induced fluorescence
